# Co-Circulation of Dengue Virus Type 3 Genotypes in Vientiane Capital, Lao PDR

**DOI:** 10.1371/journal.pone.0115569

**Published:** 2014-12-31

**Authors:** Malayvanh Lao, Valérie Caro, Jean-Michel Thiberge, Phaitong Bounmany, Khamsing Vongpayloth, Philippe Buchy, Veasna Duong, Chansamone Vanhlasy, Jean-Marie Hospied, Manichanh Thongsna, Khamla Choumlivong, Phonesavanh Vongkhamchanh, Bounleua Oudavong, Paul T. Brey, Marc Grandadam

**Affiliations:** 1 Institut Pasteur du Laos, Vientiane, Lao PDR; 2 Institut Pasteur, Paris, France; 3 Institut Pasteur du Cambodge, Phnom Penh, Cambodia; 4 Military Hospital 103, Vientiane, Lao PDR; 5 Centre Medical de l'ambassade de France, Vientiane, Lao PDR; 6 Mittaphab Hospital, Vientiane, Lao PDR; 7 Setthathirath Hospital, Vientiane, Lao PDR; 8 5 April Police Hospital, Vientiane, Lao PDR; 9 Children Hospital, Vientiane, Lao PDR; University of California Davis, United States of America

## Abstract

During the 2012 epidemic of dengue in Vientiane capital, Lao PDR, a major serotype switch from dengue 1 to 3 was observed. A molecular epidemiology study demonstrated that dengue 3 remained the predominant serotype in 2013, but also revealed the co-circulation of two genotypes, supporting the hypothesis of multiple geographic origins of dengue 3 strains circulating in Vientiane capital.

## Introduction

Dengue fever is the most important and rapidly spreading vector-borne viral disease in the tropics [1]. Despite important efforts for surveillance and prevention, large-scale epidemics frequently occur in endemic countries. Prediction of dengue epidemics based on serotype identification is a valuable approach only if a significant number of samples are analyzed over long periods of time [1], [2]. Intensive efforts to improve serotype surveillance and vector control in various islands in the Caribbean significantly improved dengue outbreak management [2]. In contrast, some South-East Asian countries, e.g., Thailand, Cambodia and Vietnam report a plethora of cases every year despite active serotype surveillance [3], [4]. In Lao Peoples' Democratic Republic, samples for dengue typing have been collected since 1987, but until now available data remained scarce [http://www.wpro.who.int/emerging_diseases/DengueWPRO20108Sep2011.pdf]. Since 2010, a robust complementary network has been set up in Vientiane capital to reinforce diagnostic capacities and to improve dengue surveillance [5]. This surveillance network allowed us to detect a switch from dengue virus (DENV) serotype 1 to 3 and to follow the progression and expansion of DENV-3 serotype and its genotypes during the 2012–2013 epidemics in Vientiane capital.

## Methods

### Sample collection

In Vientiane capital, from March 2012 to December 2013 a surveillance network, coordinated by the Institut Pasteur du Laos, investigated suspected dengue patients to determine dengue serotypes and genotypes. Suspected dengue cases were defined as patients who had undergone a medical consultation or were hospitalized in one of the partner clinical facilities in Vientiane who presented with sudden fever onset (≥38°C) for less than 7 days with at least one of the following accompanying symptoms: headache; myalgia; arthralgia; retro-orbital pain; digestive troubles or hemorrhaging. Patients included were either Lao nationals or expatriates, residing in Vientiane city or repatriated from provinces for medical reason and hospitalized in Vientiane. After obtaining informed consent, a 5 ml venous blood sample was taken from patients in hospitals or consultation facilities. Samples were stored at 4°C until transportation to the Institut Pasteur du Laos for analysis.

### Ethics statement

The study protocol was approved by the National Ethic Committee for Health Research of the Ministry of Health of Lao PDR. All public hospitals' management committees approved the study and obtained the agreement of the Ministry of health for participating with the protocol.

All adult volunteers provided a written informed consent. A parent or legal guardian of any child included in the study signed a consent form on their behalf.

### RT-PCR amplification

Samples were screened for dengue virus and serotypes were determined by a pan-dengue RT-PCR and specific real time RT-PCRs [6], [7].

Total viral RNA was extracted from the supernatant of C6/36 cultures inoculated with viremic human plasmas or directly from human plasmas. In addition, a DENV-3 strain, isolated from an autochthonous case in Vientiane capital in 2011 was analysed.

Extractions were carried out using NucleoSpin II RNA kit (Macherey Nagel) according to the manufacturer's instructions.

### Envelope gene sequencing

Sequencing of the complete envelope (E) gene (1479 nt) was performed on sixty-two Lao isolates of DENV-3 (Table 1). Amplicons were generated using the Superscript One Step kit (Invitrogen). The one-step RT-PCR reaction was performed in a volume of 25 µL containing 2 µL RNA template, 7 µL ddH2O, 12.5 µL RT-PCR buffer (5X), 1 µL sense oligonucleotide (10 µM), 1 µL anti-sense oligonucleotide (10 µM), 0.5 µL DMSO and 1 µL enzyme mix. The following set of specific primers was used to produce 3 overlapping amplicons: Den3-1F 5′-AGT TGT TAG TCT ACG TG-3′, Den3-1013R 5′ GGT AGT CAC ACA CCC CCC GTG-3′, Den3-815F 5′-GCC CTT AGG CAC CCA GGG TT-3′, Den3-1752R 5′-CCC GCG AAA ATG CTT GTG C-3′, Den3-1398F 5′-CGC AAG GAG TCA CGG CTG AG-3′, Den3-2539R 5′-GCC TGC AAT GGC TGT TGC C-3′. The amplification program was performed as follows: reverse transcription at 50°C for 30 min, an inactivation of RT enzyme step at 94°C for 2 min, followed by 35 cycles of 94°C 15 s, 55°C 30 s, 72°C 1 min 30 s, and a final step at 72°C for 10 min. PCR products were purified using the NucleoFast kit (Macherey Nagel) as specified by the manufacturer. Sequencing was carried out using the BigDye Terminator Cycle Sequencing Ready Reaction kit version 1.1 (Applied Biosystems). The sequencing reaction was performed in a volume of 10 µL containing 2 µL PCR product template, 4 µL ddH2O, 1 µL sequencing buffer (5X), 1 µL oligonucleotide (4 µM) and 2 µL Big Dye version 1.1. The sequencing program was performed as follows: 96°C 1 min followed by 30 cycles of 96°C 10 s, 50°C 5 s, 60°C 1 min 15 s. Sequence chromatograms for both strands were obtained using an automated sequence analyzer ABI3730XL (Applied Biosystems).

**Table 1:** List of studied Lao DENV-3 isolates.

Isolate ID	Village	District	Province	Date	Genotype	Acc. Number
2011-1442	Dong Naxok	Sikhotabong	Vientiane Capital	2011	II	HG530140
2012-0036	Sod	Khong	Champasak	13/06/2012	II	HG530199
2012-0039	Nonsavang	Saythany	Vientiane Capital	19/06/2012	II	HG530158
2012-0079	?	?	Northern Laos	16/07/2012	II	HG530159
2012-0093	Sednamom	Khong	Champasak	18/07/2012	II	HG530200
2012-0096	Phakao	Saythany	Vientiane Capital	17/07/2012	II	HG530160
2012-0098	Sapharmo	Saysettha	Vientiane Capital	23/07/2012	II	HG530161
2012-0099	Viengkeo	Saysettha	Vientiane Capital	19/07/2012	II	HG530162
2012-0103	Chommany	Saysettha	Vientiane Capital	22/07/2012	II	HG530141
2012-0111	Chommany	Saysettha	Vientiane Capital	24/07/2012	II	HG530142
2012-0114	Nongtha Tai	Chanthabury	Vientiane Capital	31/07/2012	II	HG530143
2012-0119	Donkoy	Sisattanak	Vientiane Capital	28/07/2012	II	HG530144
2012-0125	Donkoy	Sisattanak	Vientiane Capital	31/07/2012	II	HG530145
2012-0141	?	Saythany	Vientiane Capital	04/08/2012	II	HG530146
2012-0174	Xay	Saythany	Vientiane Capital	14/08/2012	II	HG530147
2012-0185	Huana	Numbark	Luang Prabang	18/08/2012	II	HG530148
2012-0190	Phon Kham	Sikhotabong	Vientiane Capital	17/08/2012	II	HG530149
2012-0200	Houy Hong	Chanthabury	Vientiane Capital	21/08/2012	II	HG530150
2012-0211	Ban Dounnoun	Saythany	Vientiane Capital	26/08/2012	II	HG530151
2012-0225	Phonpapao	Sisattanak	Vientiane Capital	30/08/2012	II	HG530152
2012-0211	Nonsaad	Saythany	Vientiane Capital	30/08/2012	II	HG530153
2012-0241	Chommany	Chanthabury	Vientiane Capital	06/09/2012	II	HG530154
2012-0247	Saunmome	Hatsaifong	Vientiane Capital	07/09/2012	II	HG530155
2012-0267	Veunkham	Saythany	Vientiane Capital	18/09/2012	II	HG530156
2012-0273	Sokkham	Saysettha	Vientiane Capital	14/09/2012	II	HG530163
2012-0275	Anmone	Saysettha	Vientiane Capital	18/09/2012	II	HG530157
2012-0280	Watnark	Sisattanak	Vientiane Capital	17/09/2012	II	HG530164
2012-0312	Phonsay	Saysettha	Vientiane Capital	02/10/2012	II	HG530167
2012-0318	Dongnasok	Sikhotabong	Vientiane Capital	02/10/2012	II	HG530168
2012-0319	Saynumgneun	Saythany	Vientiane Capital	02/10/2012	II	HG530169
2012-0321	Phonesavang	Sisattanak	Vientiane Capital	04/10/2012	II	HG530170
2012-0330	Chommany	Saysettha	Vientiane Capital	09/10/2012	II	HG530167
2012-0357	Aksang	Phonhog	Vientiane Province	29/10/2012	II	HG530172
2012-0358	Aksang	Phonhog	Vientiane Province	29/10/2012	II	HG530173
2012-0379	Sivilay	Saythany	Vientiane Capital	25/10/2012	II	HG530174
2012-0405	Luang Prabang	Luang Prabang	Luang Prabang	12/11/2012	II	LN680425
2012-0406	?	?	Vientiane Capital	11/11/2012	II	HG530175
2012-0409	Phonesinoun	Sisattanak	Vientiane Capital	07/11/2012	II	LN680426
2012-0411	Saynumgneun	Saythany	Vientiane Capital	08/11/2012	II	HG530178
2012-0412	?	?	Vientiane Capital	13/11/2012	II	HG530179
2012-0413	Watnark	Sisattanak	Vientiane Capital	13/11/2012	II	HG530180
2012-0428	Dongsavath	Sisattanak	Vientiane Capital	15/11/2012	II	HG530183
2012-0429	Nahai	Hatsaifong	Vientiane Capital	16/11/2012	II	HG530184
2012-0432	Khamhong	Saythany	Vientiane Capital	19/11/2012	II	HG530185
2012-0437	Done Meuy	Houe	Oudomxay	23/11/2012	II	HG530186
2012-0440	Dongpholao	Hatsaifong	Vientiane Capital	23/11/2012	II	LN680427
2012-0441	Phonsaad	Saysettha	Vientiane Capital	23/11/2012	II	HG530187
2012-0444	Choinaimo	Saysettha	Vientiane Capital	23/11/2012	II	HG530188
2012-0451	Sengsavanh	Saysettha	Vientiane Capital	28/11/2012	II	HG530189
2012-0452	Ponsavanh	Sisattanak	Vientiane Capital	27/11/2012	II	HG530190
2012-0453	Saphanmohr	Saysettha	Vientiane Capital	26/11/2012	II	HG530191
2012-0454	Ponsavanh	Sisattanak	Vientiane Capital	26/11/2012	II	HG530192
2012-0455	Khamhong	Saythany	Vientiane Capital	29/11/2012	II	HG530193
2012-0459	Nongtha Neu	Chanthabury	Vientiane Capital	28/11/2012	II	HG530194
2012-0467	Sithan Neu	Sikhotabong	Vientiane Capital	05/12/2012	II	HG530195
2012-0469	Dokdong	Saythany	Vientiane Capital	04/12/2012	II	HG530196
2012-0472	Dongmakkhai	Saythany	Vientiane Capital	09/11/2012	III	HG530197
2012-0476	Phakao	Saythany	Vientiane Capital	07/12/2012	III	HG530198
2013-0350	Dongmakkhai	Saythany	Vientiane Capital	12/01/2013	III	LN680428
2013-0557	Na Khok Noy	Saythany	Vientiane Capital	29/01/2013	II	HG530201
2013-0688	Dongmakkhai	Saythany	Vientiane Capital	31/03/2013	III	LN680429
2013-0689	Nakhouy Tai	Saysettha	Vientiane Capital	10/04/2013	II	HG530202

### Phylogenetic analysis

A total of 62 DENV-3 E gene sequences were used in this study. Nucleotides sequences of complete E genes of DENV-3 isolates used were submitted to EMBL-EBI and their accession numbers are shown in Table 1. The nucleotide sequences of complete E genes of Lao DENV isolates were aligned, edited, and analyzed using the software BioNumerics v 6.6 (Applied-Maths, Saint-Martens-Latem, Belgium).

For a comprehensive phylogenetic analysis, the data included 16 DENV-3 E sequences of Lao, PDR selected to be representative of the different provinces and covering the studied period and 25 closest reference strains of DENV-3, i.e. genotypes I, II, II and IV, obtained from GenBank.

Phylogenetic analyses, using maximum likelihood method, were conducted using MEGA version 6 (www.megasoftware.net). A maximum-likelihood tree was constructed based on the Tamura-Nei model. Sequence of DENV-3/Puerto Rico/1963 genotype IV (L11433) was used as an outgroup to root the tree. The robustness of nodes was assessed with 1,000 bootstrap replicates.

## Results

Over a period of 21 months, a total of 2780 cases with a suspected dengue infection were recruited through our clinical network in Vientiane capital. The sex ratio was 1.03∶1 (50,7% males; 49,3% females) and the median age was 20 years (range: 5 months - 78 years). Most of the patients hospitalized were residents of Vientiane capital or suburban areas. A few cases were repatriated from provinces (Northern Lao PDR; n = 1; Oudomxai, n = 1; Luang Prabang, n = 2; Champasack, n = 2).

**Figure 1:**
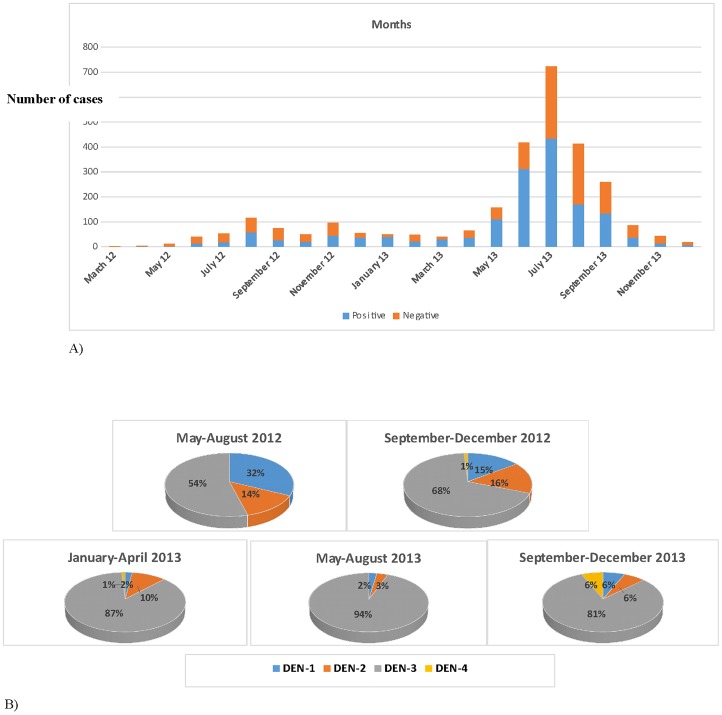
Surveillance performed by Institut Pasteur du Laos from March 2012 to December 2013. (A) Distribution of confirmed cases (i.e. RT-PCR and/or NS1 and/or culture positive) in Vientiane city (B). Dengue serotypes distribution in 2012 and 2013. Recording of dengue serotypes distribution on a four monthly basis. As the surveillance only started in late March 2012, partial data collected in April were not included in the figure.

Longitudinal analysis revealed an early and efficient transmission of dengue virus in Vientiane capital during the dry season (November-April) preceding an increase of incidence during the dry season (May-September; Fig. 1A). More importantly, severe clinical presentations including fatal confirmed cases, were recorded during the dry season (data not shown). Over the period studied, 2780 samples from patients matching the case definition were investigated. Confirmation of dengue infection could be established by RT-PCR in a total of 1535 patients (55.2%). The rate of confirmation was significantly different, varying from 44.7% (227/507) in 2012 to 57.5% (1308/2273) in 2013.

Exhaustive serotype determination by RT-PCR and/or sequencing was systematically carried out in 2012. In 2013, as the number of suspected cases in Vientiane dramatically increased, the real-time serotype surveillance was limited to a subset (≈10%) of the weekly confirmed cases.

Dengue serotype 1 (66%) and serotype 2 (33%) co-circulated from April to May 2012, but the exhaustive typing of positive samples revealed a sudden circulation and a high incidence of dengue serotype 3 in June 2012 (Fig. 1B). Since the end of June 2012, DENV-3 became the predominant serotype in circulation. DENV-3 circulation continued during the dry season (November to April 2013) with an increase in the proportion of DENV-3 up to 87%. From May to August 2013, DENV-3 incidence further increased up to 94%. DENV-4 ranged from ≤1% to 6% over the period studied.

**Figure 2:**
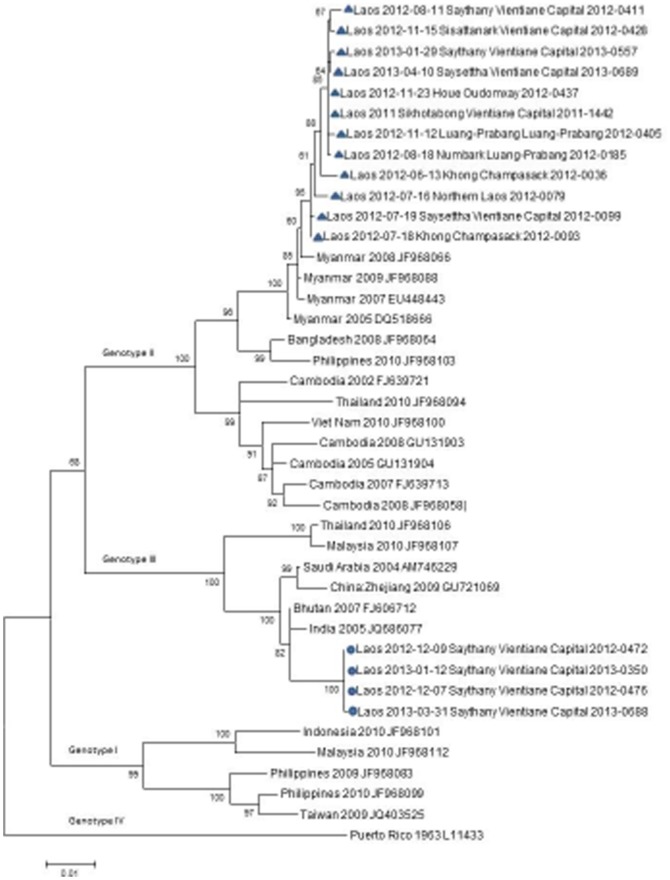
Phylogenetic relationships of DENV-3 based on the complete E nucleotide sequences (1479 nt) of 41 isolates, including 16 Lao isolates. Blue triangles and blue circles represent Lao DENV-3 genotype II and III strains respectively. Sequence alignments were performed using BioNumerics version 6.6. A maximum-Likelihood tree was constructed using MEGA version 6, based on Tamura-Nei model. Bootstrap resampling values are indicated at major nodes. Scale bar indicates number of base substitutions per site.

A total of 62 isolates were sequenced and analyzed, corresponding to various foci of transmission and during different periods of DENV-3 circulation in Vientiane capital, (Table 1). For clarity, only a subset of 16 Lao DENV-3 envelope gene sequences representative of the different provinces and covering the studied period was used for phylogenetic analysis (Fig. 2). Sequences were compared to 25 reference sequences of different DENV-3 genotypes available in GenBank.

Among the four genotypes of DENV-3 (I-IV), the Lao DENV-3 fell into genotypes II and III appearing during two distinct periods. DENV-3 genotype II viruses were detected from June 2012 onwards, whereas genotype III viruses were only isolated since November 2012.

In this series, most of the Lao viruses (58 isolates) belonged to genotype II as for other Asian isolates recently described in Cambodia, Thailand and Vietnam. These Lao isolates shared 99.5% nucleotide identity (99.6% amino-acid identity) The homogeneity of this group was independent of the date and geographic distribution (up to 1200 km from the northern province Oudomxai to Champasak province in the south) suggesting a long lasting circulation of DENV-3 genotype II.

This group of Lao isolates is strongly related to strains from Myanmar isolated between 2005 and 2009 (99.2% nucleotide identity between the two groups) and form an independent cluster well supported by a high bootstrap value.

DENV-3 genotype III Lao isolates displayed 99.93% nucleotide identity between each other, and showed a close relationship with isolates from India (2005) and Bhutan (2007). The fact that genotype III was only identified after November 2012 in Vientiane Capital supports a recent introduction in the city from another Asian country.

Our data suggested that the introduction of genotype II and III were most likely independent and singular events.

Thus, this analysis indicates that at least two genotypes of DENV-3 co-circulate in the country, corresponding to the commonly observed genotype in this geographical area, with a current the predominance of genotype II. Further investigation on DENV-3 isolates from 2013 is ongoing to follow the evolution and dynamics of these two genotypes.

In 2013, at week 52 the total number of suspected cases recorded through the syndromic surveillance system, coordinated by the Lao Ministry of Health, exceeded 48000 cases with 95 deaths. Only nine of the twenty fatal cases investigated by the Institut Pasteur du Laos were confirmed positive for dengue infection. Among those positive for DENV-3 (n = 4), genotype II was found in three patients and genotype III in one patient.

## Discussion

A dengue surveillance and alert system based on a weekly recording of dengue like syndroms has been in place in Lao PDR since 2006 but laboratory confirmation and dengue serotyping are only performed when the number of weekly cases exceeds the historical threshold. Even though a limited number of cases were analyzed yearly at the country level, this approach provided some preliminary data on predominant dengue serotypes over time [5]. However, as only few sequence data are available to date, it is nearly impossible to define the origin(s) of dengue strains, the dynamic of the serotypes and the mechanisms underlying the circulation of dengue in Lao PDR. In Vientiane capital, our network allowed following the proportions of dengue confirmed cases and of virus serotypes in real time. Initiatives of independent research groups started providing important information on dengue phylogeny [8]. Interestingly, these data obtained in a different context, (i.e. rural area, other dengue serotype 1) overlapped our observation of an active circulation of dengue virus during the dry season. From March 2013, the national network for dengue surveillance recorded a precocious and abnormally rapid increase of dengue cases in the provinces and in Vientiane capital until the epidemic subsided in October 2013. A total of 48772 dengue cases with 95 deaths were officially declared by the Lao Ministry of Health. The early and intense Southwest monsoon brought unusually heavy rains favoring the country-wide explosive emergence of dengue vector species (*Ae. aegypti* and *Ae. albopictus*), which could in part explain the importance and breadth of the 2013 epidemic. The second putative contributing parameter could have been a very low incidence of DENV-3 circulation at least during the past three years [5], [8], hence a more immunologically permissive environment for DENV-3 expansion. Retrospectively, in 2010 dengue 1 was predominant [8] whereas the year 2011 was marked by a low dengue activity. Indeed, in 2011only one DENV-3 isolate was detected in Vientiane capital through our surveillance network. Over the period studied (2010–2013), a very low genetic diversity was observed within both dengue 3 genotypes, supporting the hypothesis of a continuous local circulation of these strains. The putative introduction of DENV-3 genotype II isolates from Myanmar occurred probably before or during 2011. Genotype III was most likely introduced into Vientiane capital in late 2012 with no evidence of a previous circulation of this genotype in other Lao provinces. The patients with DENV-3 genotype III infections did not declare any travel aboard within the two weeks prior to symptom onset, but sequence comparisons support an Asian origin of the virus with close links to strains from India and Bhutan. Recent studies in India and China also point to the emergence of DENV-3 genotype III between 2009 and 2011 [9], [10]. In both cases, sequence comparison supports importation from Singapore or Vietnam, highlighting the diversity of dengue strain in Southeast Asia. Lao PDR is a landlocked country, becoming more and more a land-linked country sharing borders with five countries. This particular geographic situation may impact dengue epidemiology in that it may favor the cross boarder movement of viral isolates. A better understanding of DENV movement is now considered as a key point as emergence of new strains or genotypes have been associated with increased clinical severity [12], [13], [14]. Our study demonstrated that during the 2012–2013 epidemic, both DENV-3 genotypes II and III detected in Vientiane city were associated with death. We could not draw any statistical conclusion in terms of virulence as systematic genotyping has not yet been performed. The study of the dynamics of the two DENV-3 genotypes is still ongoing, however, it seems more likely that the serotype switch from DENV-1 to DENV-3 observed in June 2012, was the main event at the origin of the large scale 2013 epidemic in the Laotian capital. An update on the dengue surveillance in Lao PDR has been recently published [5]. Dengue serotypes proportions are provided for the last seven years at the country level, but regarding the total number of samples typed yearly, the significance of these data and their usefulness in terms of outbreak prediction are questionable. Fatal cases associated with dengue infections are rarely investigated in Laos like in most countries in Southeast Asia. Thus, the improvement of diagnostic capacities are requisite to better evaluate the real impact of dengue mortality particularly in the context of serotype switching and/or genotypes switching and viral virulence. Genotyping of dengue strains in Laos is in an early phase. Presently, there is no national biobank of isolates that could help establish the history of dengue serotypes and genotypes in Lao PDR. The identification of the co-circulation of multiple genotypes highlights the need to maintain efforts to understand the movement and dispersal of dengue strains throughout the Indochinese peninsula [3], [4], [5], [8], [9], [10], [11].
